# The Outcome and Implications of Public Precautionary Measures in Taiwan–Declining Respiratory Disease Cases in the COVID-19 Pandemic

**DOI:** 10.3390/ijerph17134877

**Published:** 2020-07-06

**Authors:** Chih-Chia Hsieh, Chih-Hao Lin, William Yu Chung Wang, David J. Pauleen, Jengchung Victor Chen

**Affiliations:** 1 Department of Emergency Medicine, National Cheng Kung University Hospital, Tainan 701401, Taiwan; hsiehchihchia@gmail.com (C.-C.H.); emergency.lin@gmail.com (C.-H.L.); 2 Waikato Management School, University of Waikato, Hamilton 3210, New Zealand; 3 School of Management, Massey University, Auckland 0745, New Zealand; D.Pauleen@massey.ac.nz; 4 Institute of International Management, National Cheng Kung University, Tainan 701401, Taiwan; victor@mail.ncku.edu.tw

**Keywords:** COVID-19, public precautionary measures, universal hygiene, mass making

## Abstract

With the rapid development of the COVID-19 pandemic, countries are trying to cope with increasing medical demands, and, at the same time, to reduce the increase of infected numbers by implementing a number of public health measures, namely non-pharmaceutical interventions (NPIs). These public health measures can include social distancing, frequent handwashing, and personal protective equipment (PPE) at the personal level; at the community and the government level, these measures can range from canceling activities, avoiding mass gatherings, closing facilities, and, at the extreme, enacting national or provincial lockdowns. Rather than completely stopping the infectious disease, the major purpose of these NPIs in facing an emerging infectious disease is to reduce the contact rate within the population, and reduce the spread of the virus until the time a vaccine or reliable medications become available. The idea is to avoid a surge of patients with severe symptoms beyond the capacity of the hospitals’ medical resources, which would lead to more mortality and morbidity. While many countries have experienced steep curves in new cases, some, including Hong Kong, Vietnam, South Korea, New Zealand, and Taiwan, seem to have controlled or even eliminated the infection locally. From its first case of COVID-19 on the 21 January until the 12 May, Taiwan had 440 cases, including just 55 local infections, and seven deaths in total, representing 1.85 cases per 100,000 population and a 1.5% death rate (based on the Worldometer 2020 statistics of Taiwan’s population of 23.8 million). This paper presents evidence that spread prevention involving mass masking and universal hygiene at the early stage of the COVID-19 pandemic resulted in a 50% decline of infectious respiratory diseases, based on historical data during the influenza season in Taiwan. These outcomes provide potential support for the effectiveness of widely implementing public health precaution measures in controlling COVID-19 without a lockdown policy.

## 1. Introduction

With the rapid development of the COVID-19 pandemic, countries are trying to cope with increasing medical demands, and at the same time, to delay the increase of infected numbers by a number of public health measures, namely non-pharmaceutical interventions (NPIs) [1]. At the personal level, actions are promoted such as social distancing, covering coughs and sneezes, frequent handwashing, and personal protective equipment (PPE); at the community level, actions may include canceling activities, avoiding mass gatherings, and closing facilities, and at the extreme, many countries have implemented lockdowns that force people to stay at home except for essential reasons. The major purpose of these NPIs in facing an emerging infectious disease is to slow the spread of the virus and suppress the reproduction of virus cases (i.e., reduce the reproduction number R, below 1) [1], until the time that a vaccine or reliable medications are available. This strategy, also described by a CDC report [2] as “flatten the curve”, is to avoid a surge of severely-ill patients that causes hospital overcrowding leading to increased mortality and morbidity, due to insufficient or overwhelmed medical resources. 

While many countries have experienced steep curves in new cases, some, such as Hong Kong, South Korea, New Zealand, and Taiwan, seem to have flattened the curve. While some successful cases have implemented lockdowns, Taiwan, with its public precaution measures, did not implement a lockdown. From its first case of COVID-19 on the 21 January until the 12 May, Taiwan had 440 cases and seven deaths in total, representing 1.85 cases per 100,000 population and a 1.5% death rate (based on the Wordometer 2020 statistics of Taiwan’s population of 23.8 million). This paper presents evidence that spread prevention involving mass masking and universal hygiene at the early stage of the COVID-19 pandemic resulted in a 50% decline of infectious respiratory diseases, based on historical data during the influenza season in Taiwan. These outcomes provide potential support for the effectiveness of widely implementing public precaution measures in controlling COVID-19, which can be transmitted by patients with respiratory symptoms, as well as with asymptomatic and pre-symptomatic carriers.

## 2. The Responses to COVID-19 in Taiwan and the Notable Decline in the Number of Respiratory Disease Cases

Taiwan was one of the first countries to respond to the pandemic. As early as the end of December 2019 when Taiwan first noticed a novel coronavirus emerging from Wuhan in China. It responded by monitoring travelers from there with enhanced measures and procedures, including the use of thermal screening systems to check suspected cases (see [3]). On the 22 January, the Taiwanese government announced increased levels of disease control, including the cessation of all tourist groups from Wuhan and the banning of exports of medical level masks (e.g., normal medical masks, surgical masks, FFP2, and N95). Such measures were supported by the literature [4], as well as by Taiwan’s experiences during SARS2003. On the 25 January, the Taiwanese National Health Insurance Administration (TNHIA) began to modify their information systems to connect to the National Customs database. Within two days, the clinical staff at medical institutes were able to see the travel history of each patient and to know instantly whether the patient had been to high-risk areas within the previous 30 days and should be under self-quarantine. This action helped medical staff identify patients with significant travel history quickly, and prevented unequipped medical staff from exposure to possible infected patients. Subsequently, just one incident involving three nurses infected via an unmasked patient due to her asthma symptom was reported in Taiwan (in Taiwan CDC COVID-19 Daily Report 29 February 2020).

Apart from all the other public health measures and NPIs [1] implemented by many countries, there are two action categories exemplified by Taiwan that seem to be unique in the beginning phase of the COVID-19 pandemic compared to the rest of the world—universal hygiene and mass masking—that could potentially influence the transmission of other infectious diseases.

### 2.1. Universal Hygiene

(1) Universal hygiene is the idea of cleaning the surface and removing fomite in the environment. It does not only refer to hand hygiene but also the places that people may accidentally touch.
(2) One of the notable responses by the Taiwan government was to let the state-owned Taiwan Tobacco and Liquor Corporation convert its alcoholic beverage factories into the production of medical-use alcohol (75%) from the 30 January. Consequently, most organizations have been able to get sufficient quantities of alcohol to deploy in every building entrance, as well as key public locations (e.g., ticket-sales windows, railway entrances, and airport customs with its fingerprint scanners).
(3) The sanitizers and 3-L containers of alcohol were then distributed strategically—with priority given to public places such as government sites, schools, metropolitan transportation systems, airports, and medical institutes—and continuously replenished. The second priority was businesses such as restaurants, factories, and supermarkets. Then, the rest was distributed to the pharmacies and convenience stores along with the surgical masks for the public to buy.
(4) The government released several types of sanitizers suitable for different organizations, which are used to conduct periodical cleaning. Clear instructions were given to the public about the proper way of disinfection, including gloving, and the sequence of cleaning (e.g., starting with stationery, furniture, the handles of doors and drawers, and then the rest of the objects that people are likely to touch).

### 2.2. Mass Masking

(1) The Taiwan government has continued to work with all manufacturers producing medical level masks to form a “national team” to extend their production capacities and consolidate distribution from private markets to state-owned channels. This policy only applies to medical masks or masks with higher protection levels. Commencing from 1.88 million masks in a day on the 24 January, the production output increased to 13 million masks a day at the end of March, and 18 million a day by May, with a production cost of USD 0.033 each.
(2) Before the medical masks were widely available to the public, the government tested different types of cloth masks and materials and published the experiment results in the media. Thus, the public knows what types of masks are more effective to filter droplets and possibly block the virus via static electricity effects. The government has encouraged the public, since late January 2020, to choose more effective masks available on the market. In addition, the government has released clear instructions of mask-wearing, e.g., its function, how to wear it properly, and maintaining mask hygiene via government websites, social media apps, and TV broadcasting.
(3) The TNHIA developed a mask distribution ‘add-on’ to their extant systems, linking with nationwide pharmacies, and implemented a system in which every resident can present their health ID card to purchase a pre-defined number of medical masks for himself/herself and a family member at USD 17 cents each (exchange rate, 12 May 2020). From early February, people were able to use their internet devices and a downloadable app to view inventory levels and locations of all pharmacies selling masks (i.e., https://mask.pdis.nat.gov.tw/). After early March, the government further launched pre-ordering systems online, and people could order their weekly allocations and pick up the masks at local convenience stores.
(4) Wearing masks in public has been commonly seen everywhere: although it is not enforced by the government, it was highly recommended. In most situations, masking has not been a requirement from the government, but nearly all public and private facilities and organizations generally expected people to wear masks before entering, particularly all medical institutes and clinics, since late January 2020. Since 1 April, it has become compulsory for people taking public transportation in response to a mildly continuing increase in infected numbers. In short, people wearing masks were commonly seen everywhere after the government raised the precaution level—from the very beginning when human to human transmission was recognized by the World Health Organization.
(5) Based on principles of reciprocity, once the inventory level of medical masks was more than sufficient for Taiwan, it then started to exchange them with countries that could supply raw materials to produce the alcohol-based sanitizers and other personal protective equipment.

## 3. Impacts and Outcome

Since the introduction of universal hygiene and mass masking, significant positive outcomes are seen in the statistics recorded by the Taiwanese Centers for Disease Control. Notably, Taiwan has not had to lock down any areas, and people continue to maintain their ordinary lives. Most interestingly, along with the relatively low rate of COVID-19 cases, national statistics of laboratory-confirmed influenza cases and the number of patients with influenza-like illness (ILI) also seem to lend support for the effectiveness of both masking and hygiene actions.

### 3.1. The Outcome in COVID-19 Mitigation 

The total confirmed COVID-19 cases in Taiwan reached 440 on 7 May, and then continued to have zero cases up until the time this paper was updated on 20 May. With a 23.8 million population, there have been 440 cases, with only 55 locally infected cases, 349 imported cases, and a navy vessel cluster of 36 cases (note: as the vessel visited overseas sites, and thus the source of infection is unknown). The figures imply that the domestic measures have been very effective, and that community transmission might not have ever occurred, with only a very few small clusters originating from imported cases.

### 3.2. The Outcome in Influenza Mitigation

Influenza cases are relevant based on our current knowledge that both COVID-19 and influenza viruses seem to have a similar route of transmission, via droplet and direct contact, while aerosol transmissions may only occur in some specific situations [5]. Therefore, we believe that the downward trend of influenza cases during the rise of COVID-19 may reflect on the effectiveness of a country’s precautionary measures against diseases transmitted via droplet and direct contact.

**Figure 1:**
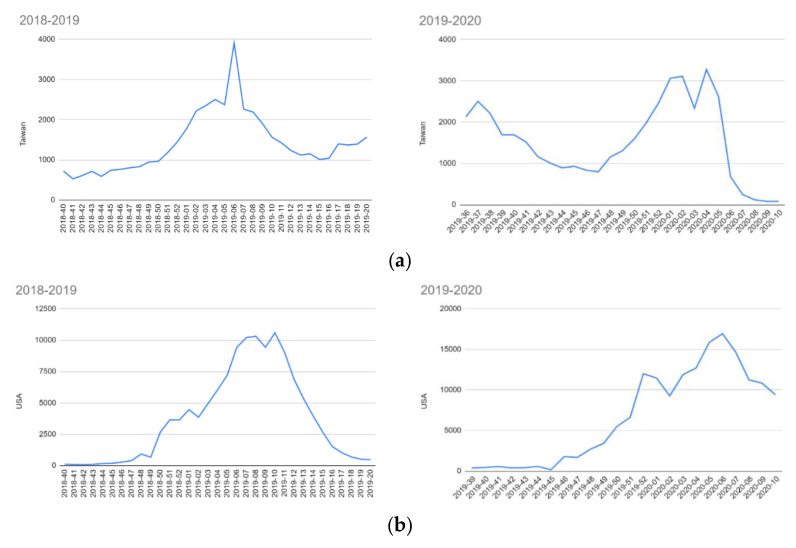
(**a**) Data extracted from Taiwanese Centers for Disease Control (Taiwan CDC) [6]. (**b**) Data extracted from American Centers for Disease Control and Prevention (US, CDC) [7]. Note: The figure shows the weekly records of confirmed influenzas A + B cases from 2018 to 2020: a comparison of cases in the US and Taiwan. *x*-axis: Year-Week interval & *y*-AXIS: number of cases.

As shown in Figure 1a,b, Taiwan has experienced a significant drop in the total confirmed cases of influenza between week 5 and week 6 of 2020, falling to a historic low point around week 9 and continuing to drop until the writing of this paper started (5 April 2020). Week 5 was from late January to early February 2020, when the government started the policy of promoting mass masking. Traditionally, February is still the influenza season. Comparative figures seen from the same period in the previous year show that they were much higher than in 2020.

Data from the same periods were gathered from the American Centers for Disease Control and Prevention, to check whether the two Northern Hemisphere countries experienced similar situations, but this was not observed. While both countries have two peaks during the 2019–2020 winter, which may be explained due to the weather conditions, the United States does not have a steep declining curve like Taiwan. Moreover, Taiwan has never had such a low number of confirmed influenza cases in February, nor such a steep rate of the drop since the national health insurance scheme was implemented in 1996.

The World Health Organization (WHO) Influenza update—368 [8] (updated on 25 May 2020) indicates the trend of the number of specimens positive for influenza by subtype. Overall, it shows that, while influenza had a dramatic drop in week 5 of 2020, it was the peak of influenza in the northern hemisphere in the WHO report.

### 3.3. The Outcome of Influenza-Like Illness (ILI)

**Figure 2:**
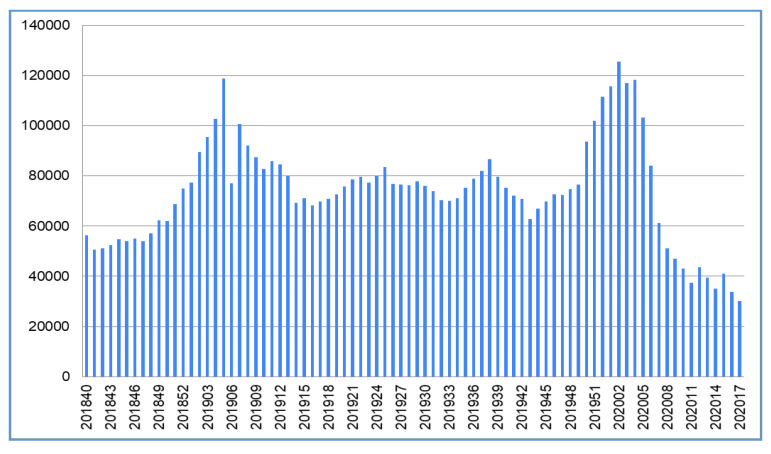
Influenza-like illness cases. Note: National Statistics of influenza-like illness (ILI), data extracted and analyzed from Taiwan CDC systems [6]. *x*-axis: year-week interval & *y*-axis: number of cases.

Apart from influenza, we checked the national statistics of influenza-like illness (ILI) from the Taiwanese Centers for Disease Control, as this may represent a wider spectrum of respiratory diseases (Figure 2 and Table 1). ILI is defined as fever (temperature of 100 °F (37.8 °C) or greater) and a cough and/or a sore throat in the absence of a known cause other than influenza by the World Health Organization [9] and adapted by Taiwanese CDC for taking cases [6]. The ILI diagram also presents the declining trend after the implementation of the public precaution measures from week 5, 2020. Three items may be worthwhile noting: (1) compared to the previous year, there are much fewer ILI cases, less than in Week 11 and Week 13; (2) the declining slope is very steep and hardly seen in the historical records, reducing by more than 40,000 cases in two weeks; and (3) Week 12 has a backward trend, with an increase of 6125 cases for one week, before dropping again in the following week. A possible explanation is that many Taiwanese overseas travelers were returning to Taiwan as the pandemic spread to many other continents. Their entering Taiwan boosted the number of confirmed COVID-19 cases from 41 on 1 March (Week 9) to 195 cases on 21 March (Week 11). ILI cases numbers are continuing to decline, and COVID-19 cases have increased at a low rate, compared to most of the other countries. A slightly higher count occurred in early April, when it was a long weekend—5 days off work when people gathered for holiday activities

## 4. Discussion and Recommendations

From the data shown above, it appears evident that the combination of mass public masking and hygiene provided a very significant result in dropping influenza and ILI at the national level. A similar outcome was also observed in South Korea, which is reported by the WHO’s Bi-weekly Influenza Situation Update. The difference is that South Korea did not report total ILI cases to WHO, but the Weekly ILI incidence rate per 1000 consultations [10]. Notably, South Korea implemented the mitigation policies several weeks later than Taiwan and this led to higher infection number in COVID-19, though the country eventually controlled it.

Initial border control and monitoring mainly targeted flights from several cities with early COVID19 outbreaks (i.e., late January to early February 2020) and, thus, border control should not generate general impacts on other transmissions of other infectious diseases at the early stage. However, it is clearly seen that the transmission of ILI and influenza were reduced right after the implementation of mass masking and universal hygiene in week 5, 2020.

Therefore, it is argued that the precautionary measures are the reasons for the outcome. Apart from these two major measures, there have not been other major measures observed/implemented to influence ILI transmissions in Taiwan. For example, contact tracing is considered a very useful prevention tool for COVID-19 [11] but tracing COVID-19 patients might not be affected by the transmission links of other ILIs. Influenza can be transmitted in the route of the droplets. Qualified surgical masks have a level of protection to filter pathogens [12]. Moreover, masks can prevent droplets from spreading from asymptomatic and, presymptomatic carriers, or patients with fewer symptoms (i.e., no respiratory symptoms). Studies have shown that patients without symptoms can shed a high level of virus load [13,14]. Potentially, transmissions from contracting the virus from the environment can be avoided, as indirect COVID-19 transmission has been reported [15].

There is a perception that Asian people tend to wear masks at greater levels and have a higher acceptance rate. This was not the case before SARS 2003. The most serious cluster transmission that occurred in Taiwan during SARS2003 was also a nosocomial infection. It did not start due to clinical activities in the theater, but rather through a symptomless laundry worker who brought the virus into the hospital and caused infections of other medical staff, which eventually led to a long term lockdown of the hospital [16]. The subsequent distributions of the virus to other hospitals were all caused by similarly unexpected people. The experience of emergency response to SARS2003 is the main reason that the Taiwan government has been highly aware of coronavirus disease, and the importance of reserving masks inventories, production capacities, and the implementation of mass masking at the government level, as well as the general awareness and acceptance of such actions by the Taiwanese public.

While SARS was contained at the cluster level in 2003, COVID-19 is more contagious and has spread much wider (SARS2003: 8096 infected, 774 dead worldwide; [17]). The phenomenon of influenza and ILI cases dropping in Taiwan seems to be significantly aligned with public precaution measures, particularly when everyone wears a mask and there are alcohol-based sanitizers at the entrances of nearly all businesses, buildings, types of public transport, dining areas and other key places people may gather.

Based on the experiences of Taiwan and the findings presented in this paper, the authors recommend the following:

(1) If community transmission occurs, mass masking for everyone, with prioritization of mask levels as a compromise between a severe societal lock-down and uncontrolled freedom. High priority should be given to healthcare workers, patients, and any staff working in medical institutions.
(2) Masking for other public sector employees working on the frontlines—police, postal delivery, receptionists of public services, schoolteachers, supermarket checkouts, food delivery, etc.
(3) Lastly, masking may be recommended to the public with caveats. If there are insufficient supplies in the market or via government sources, it may be a good idea to provide guidance on making good quality masks with materials on hand, or how to select non-medical masks with high-level filtering materials (e.g., the early stage of Taiwan, Hong Kong, South Korea, and the Czech Republic). This would be better than nothing, as masks are scientifically proven as effective in preventing droplet spreading in hospital settings [18] and surgical masks can be potentially more effective in stopping COVID-19 spread via the droplets similar to influenza [19]. Homemade masks have also been proven to be useful and recommended in the literature, though offering less protection than surgical quality masks [20,21]. Masking not only helps to stop the spread of COVID-19, but may also reduce the spread of other diseases so that valuable medical resources can be conserved.
(4) As the pandemic is continuing to develop, it is suggested that more proactive governmental interventions be considered, in order to avoid the negative impacts of a complete lockdown. This may include allocating the resources of stated-owned businesses to produce medical resources and implement anti-pandemic measures.
(5) Before a vaccine is available, governments may want to continue to increase the production/procurement capacities of masks, sanitizers, and other PPEs to supply the health institutes, and to help society as a whole to reduce all transitive respiratory diseases. This will not only slow the spread of COVID-19, but also influenzas and other flu-like illnesses. This can help reduce the overloading of medical resources and allow medical institutes to focus more on controlling the COVID-19 pandemic.
(6) Compared to other success stories, such as New Zealand and Vietnam, there has not been a lockdown in Taiwan. It makes Taiwan’s experiences even more valuable, since countries would need to prepare for the future waves of COVID-19, and lockdown can lead to huge economic costs and potentially other public health issues.

## 5. Limitations and Other Assumptions

**Figure 3:**
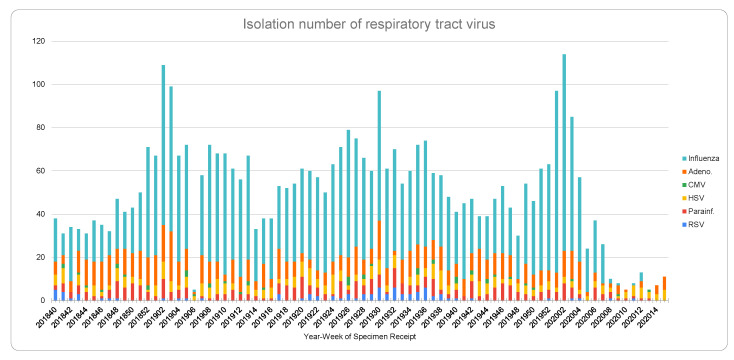
Isolation number of respiratory tract virus. Note: year-week specimen receipt from randomly selected samples of respiratory disease, data extracted from the Taiwan National Infectious Disease Statistics System [6]. It shows the number of isolates representing the distribution of most commonly seen viral infection cases selected from ILI cases. It is the aggregation of two nationwide randomly selected samples from each of the 160 sample taking stations. *x* axis: year-week interval & *y* axis: number of cases.

The positions put forth in this paper and the outcomes stated may be subject to limitations, as follows:

(1) As only two years of data were available from the United States CDC, and due to the limitation of data sources, only two years of influenza data are reported in this paper. As observed, Taiwan did not have low influenza cases in February/March in the previous years, which are considered the traditional influenza seasons. The WHO has reported week 5 is the peak of influenza cases in the Northern Hemisphere [8]. However, the report is based on specimen samples, rather than cases of the total population, as in Taiwan’s report.
(2) As this is an ongoing pandemic disease, the worldwide trajectories for countries can still change, pending the development of public health policies and public health measures in each country. Evidence supports the outcome of Taiwan’s action items in the first two months, late January to late April, during the COVID-19 pandemic in 2020.
(3) A unique situation may not have been considered yet. Taiwan is one of the countries which has a very high rate of myopia, e.g., around 84% of high school students [22], and the group with presbyopia means roughly 40 to 50% of all citizens wear glasses. The combination of eyewear, medical masks, and frequent hygiene might play a synergistic role in preventing the spread of COVID-19. As suggested by the American Academy of Ophthalmology (https://www.aao.org) and the literature analysis [23], coronavirus can potentially spread through the eyes, and, thus, replacing contact lenses with glasses is encouraged
(4) There may be changing behaviors regarding going to medical institutes during the COVID-19 pandemic. However, from Figure 3, it can be seen that there are still Herpes Simplex Virus (HSV) positive cases. It is, again, further proof that the drop of influenza and other viral species that have a similar transmission channel to COVID-19 is significant after week 5, 2020.
(5) The paper does not show direct evidence of how COVID-19 was prevented by the public health measures. However, if the transmission links of COVID-19 and influenzas/ILI are similar, the outcome from influenza and ILI, the drastically declining cases, can be seen as strong evidence and Taiwan maintains a very low local transmission of COVID-19. Moreover, the declining trends of influenzas/ILI have helped the medical institutions in reserving valuable medical resources, such as testing capacity, staff, intensive care unit (ICU) wards, ventilators in preventing and treating COVID-19 cases. There are other countries with dropping ILIs, and this brief report mainly discusses the single case of a country: it does not investigate countries that implemented policies from mild economic restrictions to complete lockdown. Future studies may highlight the levels of lockdown and their impacts on ILI mitigation.

## Figures and Tables

**Table 1:** Taiwan influenza-like illness 2020 cases during mass masking and 2019 comparison, data extracted and analyzed from Taiwan CDC systems.

Year/WeeK	Cases	Year/Week	Cases	Ratio 20/19	Week Decrease
201905	118,767	202005	103,035	0.8675	-
201906	77,109	202006	84,078	1.0904	18,957
201907	100,572	202007	61,313	0.6096	22,765
201908	92,081	202008	52,676	0.5721	8637
201909	87,515	202009	46,938	0.5363	5738
201910	82,755	202010	43,063	0.5204	3875
201911	85,730	202011	37,277	0.4348	5786
201912	84,493	202012	43,402	0.5137	−6125
201913	79,924	202013	38,270	0.4788	5132
201914	69,388	202014	34,685	0.4988	3585
